# Parasitoids of Insect Pests Feeding on *Scaevola taccada* (Goodeniaceae) from Yongxing Island in South China Sea

**DOI:** 10.3390/insects15120926

**Published:** 2024-11-26

**Authors:** Huayan Chen, Cornelis van Achterberg, Yang Li, Zhen Liu, Jun Wang, Shixiao Luo

**Affiliations:** 1 Guangdong Provincial Key Laboratory of Applied Botany, South China Botanical Garden, Chinese Academy of Sciences, Guangzhou 510650, China; huayanc@scbg.ac.cn (H.C.); wxj@scbg.ac.cn (J.W.); 2 State Key Laboratory of Plant Diversity and Specialty Crops, South China Botanical Garden, Chinese Academy of Sciences, Guangzhou 510650, China; 3 South China National Botanical Garden, Guangzhou 510650, China; 4 State Key Laboratory of Rice Biology and Ministry of Agriculture/Key Lab of Agricultural Entomology, Institute of Insect Sciences, Zhejiang University, Hangzhou 310058, China; kees@vanachterberg.org; 5 Sichuan Provincial Key Laboratory for Development and Utilization of Characteristic Horticultural Biological Resources, College of Chemistry and Life Sciences, Chengdu Normal University, Chengdu 611130, China; xly105@163.com; 6 Zoology Key Laboratory of Hunan Higher Education, College of Life and Environmental Sciences, Hunan University of Arts and Science, Changde 415000, China; qingniao8.27@163.com

**Keywords:** biological control, natural enemies, DNA barcoding, parasitism

## Abstract

*Scaevola taccada* (Goodeniaceae) is an important evergreen coastal plant on islands in the South China Sea, which shows excellent tolerance for salty and drought conditions. Nevertheless, the growth of *S. taccada* populations on these islands in the South China Sea has been threatened by a few serious insect pests. However, we know little about the biology of these pests. In this study, we surveyed and identified the parasitoids of two main pests (*Herpetogramma submarginale* (Swinhoe, 1901) and *Ophiomyia scaevolana* Shiao and Wu, 1996) of *S. taccada* communities on Yongxing Island in the South China Sea, with the aim to assess their potential in biological control. *Dolichogenidea stantoni* (Ashmead, 1904) is a gregarious endoparasitoid of the larva of *H. submarginale* and contributes an average 48.9% parasitism rate on *H. submarginale*. *Opius biroi,* Fischer, 1960 and *Euderus albitarsis* (Zetterstedt, 1838) are both solitary endoparasitoids of the larva of *O. scaevolana*, with a respective 5.8% and 64.4% parasitism rate on *O. scaevolana*. We summarize the species diagnosis, biology, and distribution of the three parasitoid species. The potential of these parasitoids used in biological control is also discussed.

## Simple Summary

The beach naupaka, *Scaevola taccada,* is an important evergreen shrub on the islands in the South China Sea because it is one of the most common plant species inhabiting those small islands and plays an important role in vegetation restoration. However, the growth of this shrub on islands in the South China Sea has been seriously damaged by a few insect pests. In this study, we investigated the natural enemies of two main pests (*a* leaf borer and a leafminer) of the beach naupaka and assessed their potential for biological control of the pests. A braconid wasp, *Dolichogenidea stantoni,* killed almost half of the leaf borers, while a eulophid wasp, *Euderus albitarsis,* killed more than half of the leafminers. Therefore, these two parasitic wasp species have great potential to be used as biological control agents to reduce the damage caused by the two main pests of the beach naupaka.

## 1. Introduction

The beach naupaka *Scaevola taccada* (Gaertn.) Roxb. (Goodeniaceae) is a perennial evergreen shrub or small tree commonly inhabiting almost the entire tropical and subtropical coastline of the Pacific and Indian Oceans [1,2]. Due to its strong adaptability to coral island environments, such as drought, salinity, and barrenness, *S. taccada* plays an important role in windbreak, sand fixation, and vegetation restoration of islands and coastal zones and has been widely used for preventing coastal erosion, for soil reclamation, and coastal landscaping [3,4].

Although unthoroughly investigated, a few insect pest species (Table S1) have been reported to feed on *S. taccada*, including leafminers [5,6,7], sawflies [7], beetles [7], scale insects [8], and thrips [9]. A pest survey and safety assessment study conducted on five islands of the Yongle Archipelago in the South China Sea found that *Ophiomyia scaevolana* Shiao and Wu, 1996 (Diptera, Agromyzidae) (misidentified as *Liriomyza sativae* Blanchard, 1938), *Arge geei,* Rohwer, 1912 (Hymenoptera, Argidae), *Phaedon brassicae,* Baly, 1874 (Coleoptera, Chrysomelidae), and *Colaphellus bowringi* (Baly, 1865) (Coleoptera, Chrysomelidae) caused very serious damage on *S. taccada* [7]. As the study suggests, these pests can easily cause serious problems, such as biosecurity and ecological environment security on the islands [7]. Currently, no specific pest management methods for *S. taccada* have been reported on the islands in the South China Sea, mainly due to the limited pest control resources available and the large population of *S. taccada* on these remote islands, although chemical pesticides are occasionally used to suppress the outbreak of insect pests (based on personal communication with local islanders). Given the importance of *S. taccada* in the vegetation restoration on islands and the vulnerable environment of those islands, sustainable pest management strategies, such as biological control using natural enemies, should be a favorable and cost-effective option. However, natural enemies of insect pests infesting *S. taccada* have not been investigated, nor have the application of natural enemies in the biological control against *S. taccada* pests.

**Figure 1:**
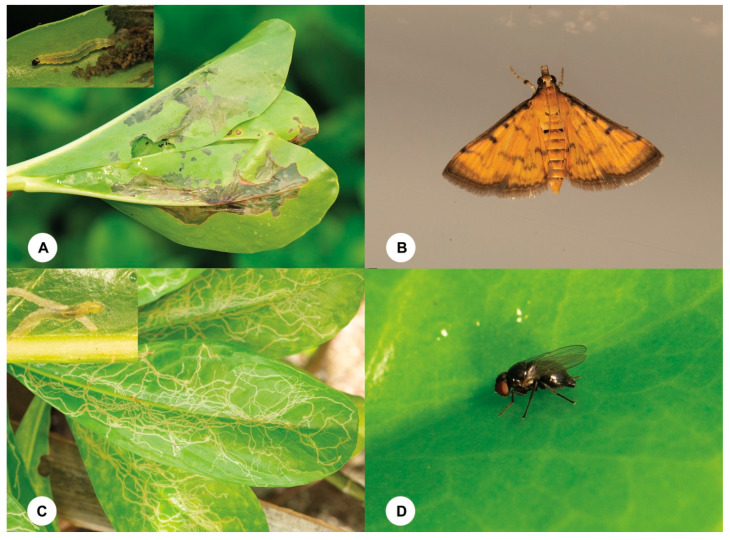
(**A**) Leaves of *Scaevola taccada* damaged by *Herpetogramma submarginale* larva. (**B**) *Herpetogramma submarginale*, female adult. (**C**) Leaves of *Scaevola taccada* damaged by *Ophiomyia scaevolana* larva. (**D**) *Ophiomyia scaevolana*, female adult.

During an expedition to Yongxing Island to survey natural enemies of insect pests, we found that *S. taccada* were heavily damaged by two insect pests: a leaf borer *Herpetogramma submarginale* (Swinhoe, 1901) (Lepidoptera, Crambidae) and a leafminer *Ophiomyia scaevolana* Shiao and Wu, 1996 (Diptera, Agromyzidae) (Figure 1). Leaves damaged by *H. submarginale* wilted, which can lead to the death of branches. Badly infested leaves by *O. scaevolana* became yellow and dropped off the plants. The growth of some *S. taccada* trees was apparently affected by these two pests (based on personal observation). The larvae of these two insect pests were also found to be attacked by parasitoid wasps. Thus, this study aims to identify and assess the potential of parasitoid species for biological control of key pests that feed on *S. taccada* on Yongxing Island in the South China Sea.

## 2. Materials and Methods

### 2.1. Insect Collection and Rearing

The field survey was conducted in January 2024 on Yongxing Island (16.83° N, 112.34° E, ca. 5 m a.s.l.), South China Sea. The identity of *H. submarginale* was confirmed by a Lepidoptera expert (Dandan Zhang, Sun Yat-sen University) using the description provided by Wang et al. [10]. The identity of *O. scaevolana* was confirmed by a Diptera expert (Li Shi, Inner Mongolia Agricultural University) using the description provided by Shiao and Wu [5]. The larvae of *H. submarginale* were randomly collected from the infested leaves of *S. taccada* and were placed individually in a plastic petri dish (100 mm × 15 mm). The larvae were fed with fresh leaves of the *S. taccada* and checked daily for the emergence of parasitoids or the pupation of *H. submarginale*. The parasitoids that emerged from each caterpillar were preserved in a 1.5 mL tube with 100% ethanol until further morphological and molecular analyses. The number of parasitized larvae, the number of parasitoids that emerged from each caterpillar, and their sex ratio were recorded.

The larva (maggot) of *O. scaevolana* burrows into the leaves of *S. taccada* to feed at night, producing narrow winding mines in the leaf but hiding at daytime in the midvein of the leaf (based on personal observation). The leaves with new mines were randomly collected and brought back indoors for dissection. The midvein of the infested leaf was cut open with a cutter knife. The larvae or pupae of parasitoids beside an infested maggot were placed individually in a 10 cm glass tube and checked daily for the emergence of parasitoids. The maggots of *O. scaevolana* without symptoms of parasitism were also placed individually in a plastic petri dish (100 mm × 15 mm) and fed with fresh leaves of *S. taccada* and checked daily for the emergence of parasitoids or the pupation of *O. scaevolana.* The number of infested maggots, the number of parasitoids that emerged from each maggot, and their sex ratio were recorded.

The larvae of *H. submarginale* and leaves infested by *O. scaevolana* were collected from three spots at Yongxing Island, and 30 larvae and 30 leaves were collected from each spot.

### 2.2. Species Identification

Parasitoid species were first determined based on morphology. The identity of the Braconidae species was confirmed by authors CvA, YL, and ZL (Braconidae taxonomy expert) using the descriptions provided by Nixon [11] and Fischer [12]. The identity of the Eulophidae species was confirmed by HYC using the key and illustration provided by Askew [13]. To supplement morphological identification, we amplified the “barcode” region of the mitochondrial cytochrome oxidase subunit 1 (COI) using a non-destructive DNA extraction protocol, as described in Taekul et al. [14]. Genomic DNA was extracted from a female and a male of each parasitoid species using a TIANamp Micro DNA Kit (Tiangen Biotech, Beijing, China). The sequences of the *COI* gene were acquired as described by Yan et al. [15]. Briefly, the *COI* gene was amplified by polymerase chain reactions (PCRs) using the LCO1490/HCO2198 primer pair [16]. Amplicons were directly sequenced in both directions with forward and reverse primers using an Applied Biosystem (ABI) 3730XL from TsingKe Biological Technology (Beijing, China). Chromatograms were assembled into contigs using Geneious 11.0.3. The assembled sequences were translated to amino acids using the invertebrate mitochondrial code to check for stop codons and frameshifts and were blasted against the GenBank database to check for identification. All sequences generated in this study were deposited in the GenBank under accession numbers listed in Table S2. Voucher specimens are deposited in the insect collection of the South China Botanical Garden, Chinese Academy of Sciences, Guangzhou, China (SCBG).

### 2.3. Imaging

Photographs of live insects were taken using a Canon 5D Mark IV (Tokyo, Japan) camera with a 100 mm macro lens. Multifocal images of mounted specimens were made using a Nikon SMZ25 microscope with a Nikon DS-Ri 2 digital camera system (Nikon Corporation, Tokyo, Japan). Images were post-processed with Adobe Photoshop CS6 Extended (Adobe Inc., San Jose, CA, USA).

### 2.4. Data Analysis

Parasitism rate (%) = (number of parasitized host larvae/total number of host larvae) × 100.

The mean and standard deviation of the parasitism rate and the female ratio were calculated using SPSS software (version 25.0, IBM Corp., Armonk, NY, USA) based on the averages derived from the three sampling points.

## 3. Results

**Figure 2:**
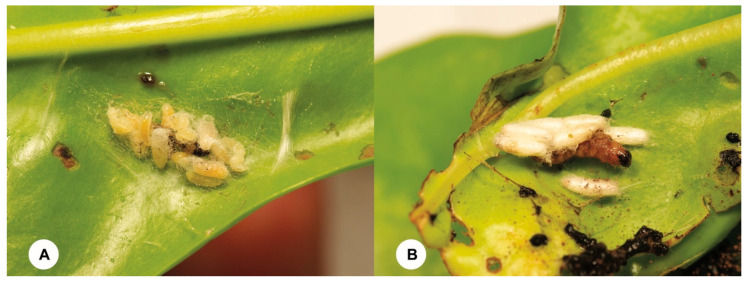
The *Dolichogenidea stantoni* (Ashmead) larvae emerged from a larva of the *Herpetogramma submarginale* caterpillar. (**A**) The parasitoid larvae begin to pupate on the host caterpillar. (**B**) The parasitoid cocoons surrounding the dead host caterpillar.

**Figure 3:**
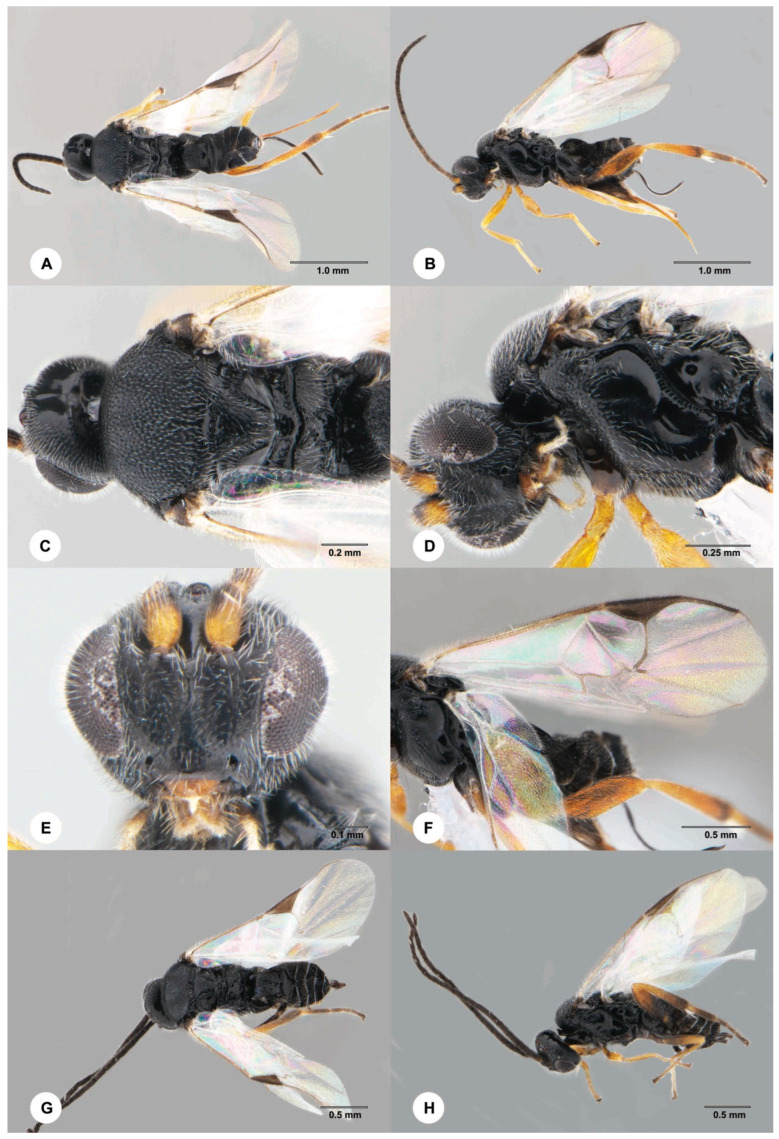
*Dolichogenidea stantoni* (Ashmead), (**A**–**F**) female. (**A**) Dorsal habitus. (**B**) Lateral habitus. (**C**) Head and mesosoma, dorsal view. (**D**) Head and mesosoma, lateral view. (**E**) Head, anterior view. (**F**) Wings (**G**,**H**) male. (**G**) Dorsal habitus. (**H**) Lateral habitus.

Of the 90 larvae of *H. submarginale* collected from three spots on Yongxing Island, 44 larvae (Table S3) were parasitized by one gregarious endoparasitoid species, *Dolichogenidea stantoni* (Ashmead, 1904) (Hymenoptera, Braconidae) (Figure 2 and Figure 3), resulting in an approximate 48.90 (± 8.4)% parasitism rate (Table 1). The number of emerged *D. stantoni* adults from each infested caterpillar ranges from 6~19, with 56.67 (±2.08)% being females (Table 1 and Table S3).

**Figure 4:**
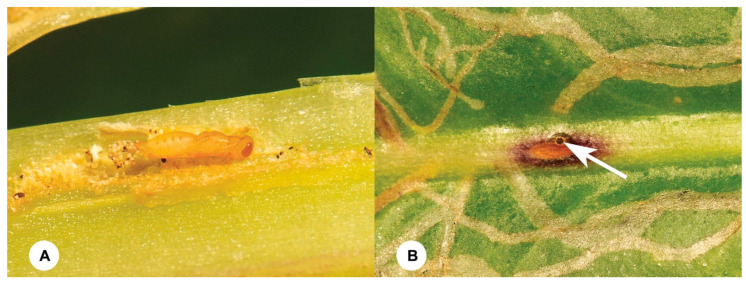
*Opius biroi* Fischer. (**A**) Pupa. (**B**) Emergence hole (white arrow).

**Figure 5:**
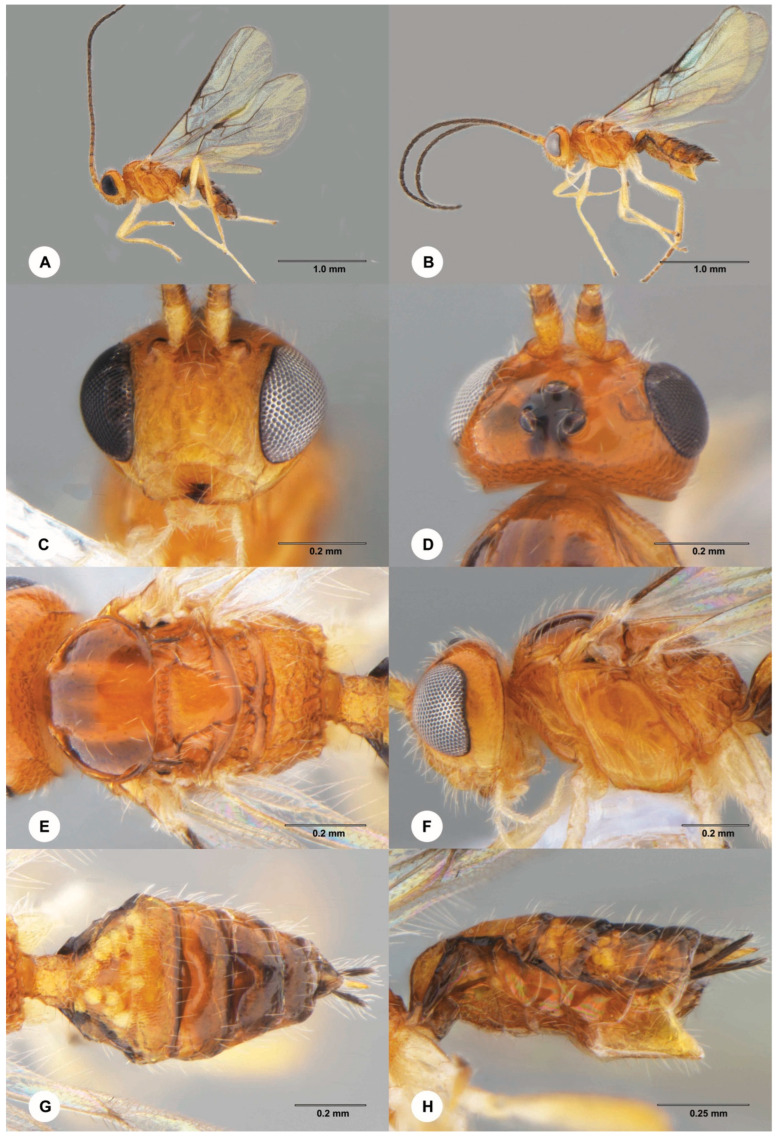
*Opius biroi* Fischer, (**A**) Male, lateral habitus. (**B**–**H**) Female, (**B**) Lateral habitus. (**C**) Head, anterior view. (**D**) Head, dorsal view. (**E**) Mesosoma, dorsal view. (**F**) Head and mesosoma, lateral view. (**G**) Metasoma, dorsal view. (**H**) Metasoma, lateral view.

**Figure 6:**
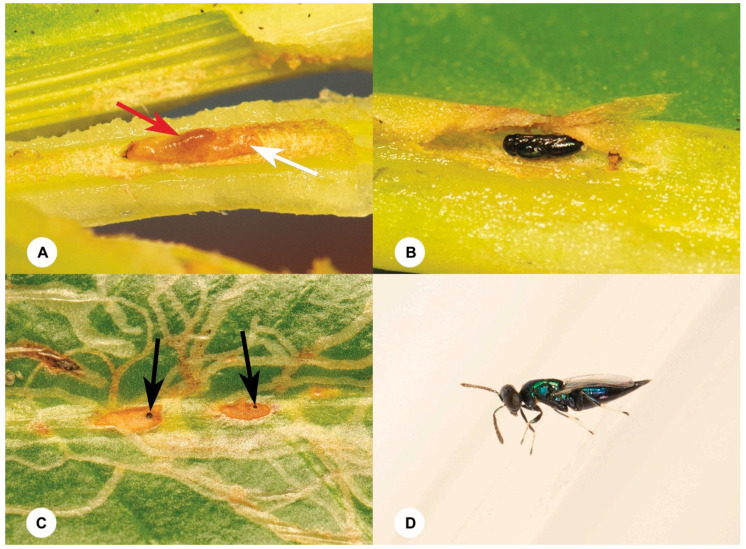
*Euderus albitarsis* (Zetterstedt). (**A**) A larva during the last instar (red arrow) feeds as an ectoparasitoid on the larva (white arrow) of *Ophiomyia scaevolana,* Shiao and Wu. (**B**) A pupa. (**C**) An emergence hole (black arrow). (**D**) A female adult.

**Figure 7:**
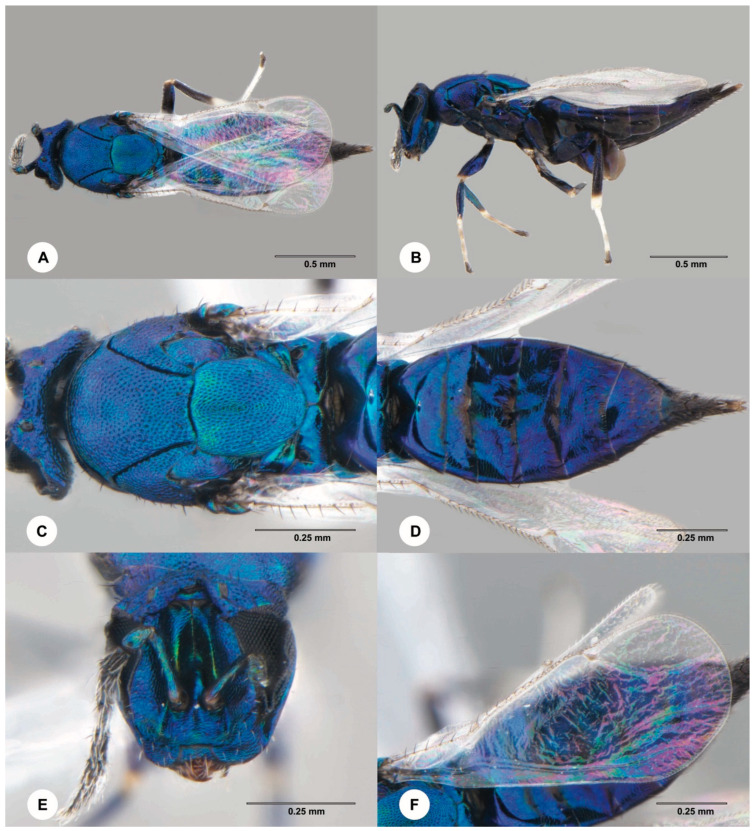
*Euderus albitarsis* (Zetterstedt), female. (**A**) Dorsal habitus. (**B**) Lateral habitus. (**C**) Head and mesosoma, dorsal view. (**D**) Metasoma, dorsal view. (**E**) Head, anterior view. (**F**) Forewing.

Of the 90 leaves infested by *O. scaevolana*, each leaf contains 2–4 infested or healthy maggots. From within parasitized maggots emerged two solitary endoparasitoid species, *Opius biroi,* Fischer, 1960 (Hymenoptera, Braconidae) (Figure 4 and Figure 5) and *Euderus albitarsis* (Zetterstedt, 1838) (Hymenoptera, Eulophidae) (Figure 6 and Figure 7). *Opius biroi* contributes an approximate 5.80 (±1.15)% parasitism rate, with 67.23 (±7.51)% females, while *E. albitarsis* is the dominant parasitoid of *O. scaevolana*, contributing an approximate 64.40 (±1.61)% parasitism rate, with 55.57 (±6.81)% females (Table 1 and Table S4).

The three parasitoid species are summarized below.

*Dolichogenidea stantoni* (Ashmead, 1904) (Braconidae)

**Diagnosis**. The female body length is about 2.5 mm. The body is black, the hind femur is yellow, the wings are hyaline, and the venation is proximal to the areolet, which is almost colorless. The propodeum has sharp and distinct areolation, and three posterior fields that are polished and smooth. The horizontal part of the first tergite is slightly transverse, being fully as long as wide, parallel-sided, and distinctly sculptured but often markedly smoother towards the apex. The second tergite has a weaker sculpture than the middle of the first tergite and is about half as long as the third tergite, and its medial area is as wide as the apex of the first tergite. The ovipositor sheath is about 1.3× longer than the hind tibia. The male is similar to the female, except for having a longer antenna (distinctly longer than the body, and the penultimate segment is 2.1× longer than wide). The first tergite has a weaker sculpture posteriorly than the female, and the second tergite is 2.8× wider than its midlength. **Note:** The identity of our specimens was mainly confirmed based on the descriptions provided by Nixon [11], but descriptions provided by Ashmead [17] and Wilkinson [18] were also compared. There are four COI sequences (Accession numbers: KJ564279, JQ849642, JQ848835, and JQ847315) labeled as *D. stantoni* present in the GenBank. The sequences of JQ849642, JQ848835, and JQ847315 match with the two sequences (Accession numbers: PQ530501 and PQ530502) we obtained in this study, with sequence similarity ranges between 98.2% and 100%. We compared our specimens with the images of the voucher specimens of JQ849642, JQ848835, and JQ847315 and found that they are highly similar. KJ564279 represents another species different from JQ849642, JQ848835, and JQ847315, and our sequences (PQ530501 and PQ530502) because of low sequence similarity (<92.3%), but the identity of this sequence cannot be confirmed because no voucher specimen is available.

**Biology**. The known hosts include: *Agonoxena pyrogramma* Meyrick (Agonoxenidae), *Cydalima laticostalis* (Guenée) (Crambidae), *Diaphania glauculalis* (Guenée) (Crambidae), *Diaphania indica* (Saunders) (Crambidae), *Diaphania pyloalis* Walker (Crambidae), *Glyphodes vertumnalis* Guenée (Crambidae), *Haritalodes derogata* (F.) (Crambidae), *Olene mendosa* (Hübner) (Erebidae)*, Olethreutes codonectis* Meyrick (Tortricidae), *Palpita marginata* Hampson (Crambidae), *Parotis marginata* (Hampson) (Crambidae), *Pieris ida* Cramer, and *Tinea pachyspila* Meyrick (Tineidae) [19]. However, some of the above host records should be viewed with some caution as previous researchers had different opinions. For example, Wilkinson recorded *D. stantoni* as a solitary parasitoid of *D. glauculalis* (= *Margaronia glauculalis* (Guenée) [18]), and Nixon considered it an erroneous record [11]. *Herpetogramma submarginale* is recorded as its host for the first time. It is a gregarious endoparasitoid of the larva of *H. submarginale*, with multiple individuals emerging from a single host.

**Distribution**. Their distribution includes China (Zhejiang, Fujian, Guangdong, Hainan, Guangxi, Guizhou, and Taiwan), Papua New Guinea, Fiji, India, Malaysia, the Philippines (type locality), and Vietnam [19].

*Opius biroi* Fischer, 1960 (Braconidae)

**Diagnosis**. The female body length is 1.5–2.0 mm. The body is mostly yellowish brown, but the stemmaticum, antenna (except for a largely brownish-yellow basal four segments), mandible apically, mesoscutum medio-anteriorly and laterally, tarsal claws, fifth to seventh tergites, and ovipositor sheath are blackish-brown. The wings are hyaline, and the pterostigma and veins are dark brown. Their antenna has 23–27 segments. The occipital carina is moderately far from the hypostomal carina and mediodorsally absent. The frons and the face are smooth. The clypeus is flattened in the lateral view, depressed ventrally, and sickle-shaped. The malar suture is distinct and linear. The pronope is medium-sized and round. The precoxal sulcus is indistinct and is as smooth as the remainder of the mesopleuron. The notauli is almost completely absent on the disc and is only anteriorly shallowly impressed. The propodeum glabrous’ surface is largely rugose, with a few crenulae posteriorly and a medio-longitudinal carina absent. The hind femur is about 4.0 times as long as it is wide, and the hind tibia is without an oblique carinula, basally. The vein SR1 of the fore wing is 3.0–3.4 times as long as vein 3-SR. The vein 1r-m of the hind wing is distinctly oblique and is 0.4 times the length of the vein 1-M. The basal cell of the hind wing is very narrow. The first tergite is as long as its apical width or slightly longer; its surface is medially smooth, slightly convex posteriorly, and rugose, and its dorsal carinae are developed, and the dorsope is absent. The second and third tergites are finely coriaceous and medio-anteriorly more or less rugulose, with the tergites being smooth. The length of the setose part of the ovipositor sheath is 0.3 times the length of the hind tibia. The male is similar to the female. **Note:** No COI sequences of this species are available in GenBank, and we here provide the first COI sequences (Accession numbers: PQ530503, PQ530504) for this species.

**Biology**. Of the solitary endoparasitoid of the larvae of several leafminer fly species with a final ectoparasitoid phase, a single individual emerged from a single host. The known hosts include *Chromatomyia horticola* (Goureau) (Agromyzidae), *Liriomyza bryoniae* (Kaltenbach) (Agromyzidae), and *Liriomyza trifolii* (Burgess) (Agromyzidae) [20]. *Ophiomyia scaevolana* is recorded as its host for the first time.

**Distribution**. It is distributed in China (Beijing, Fujian, and Guizhou) [21], Hungary [22,23], Iran [24], Italy [25], Spain (including the Canary Islands) [26,27,28], and Turkey [29].

*Euderus albitarsis* (Zetterstedt, 1838) (Eulophidae)

**Diagnosis**. The female body length is 2.1–2.4 mm. The body is violet with metasoma, which is nearly black ventrally. The antenna is violet, becoming darker apically. The legs are violet to nearly black, except for the tip of the tibiae and the first three tarsi, which are white. The wings are hyaline, and the pterostigma and veins are light brown. The female antenna with clava is only slightly broader than the fourth funicle segment. The male antenna with setae is less outstanding, rather thicker and darker. The middle lobe of the mesoscutum has six adnotaular setae. The propodeum weakly reticulates with three setae on the callus, one-fourth as long as the scutellum. The forewings have four apical setal lines. The postmarginal vein is shorter than 1.5 times that of the stigmal vein. The female metasoma lanceolate exerted ovipositor sheaths longer than the last tergite propodeum, with three setae on the callus. **Note:** The COI sequences (Accession numbers: PQ530505 and PQ530506) we attained highly matched (around 99% similarity) with four sequences (Accession numbers: MG836466–MG836469) labeled as *E. albitarsis* in the GenBank.

**Biology**. Of the solitary endoparasitoid of larvae belonging to the four orders of insects, one single individual emerged from a single host. The known hosts include *Saperda populnea* (Linnaeus) (Coleoptera, Cerambycidae)*, Ceutorhynchus constrictus* Schoenherr (Coleoptera, Curculionidae), *Ceutorhynchus obstrictus* (Marsham) (Coleoptera, Curculionidae), *Scolytus intricatus* (Ratzeburg) (Coleoptera, Curculionidae), *O. scaevolana* (Diptera, Agromyzidae), *Asphondylia pictipennis* Kieffer (Diptera, Cecidomyiidae), *Hartigia xanthostoma* (Eversmann) (Hymenoptera, Cephidae), *Phylloecus linearis* (Schrank) (Hymenoptera, Cephidae), *Zeuzera pyrina* L. (Lepidoptera, Cossidae)*, Lymantria monacha* Moore (Lepidoptera, Erebidae), *Ectoedemia sericopeza* (Zeller) (Lepidoptera, Nepticulidae), *Trifurcula sericopeza* (Zeller) (Lepidoptera, Nepticulidae), *Thaumetopoea pityocampa* Denis and Schiffermüller (Lepidoptera, Notodontidae), *Adaina microdactyla* (Hübner) (Lepidoptera, Pterophoridae), *Rhyacionia buoliana* (Denis and Schiffermüller) (Lepidoptera, Tortricidae), and *Spilonota ocellana* (Denis and Schiffermüller) (Lepidoptera, Tortricidae) [30,31,32]. *Ophiomyia scaevolana* is recorded as its host for the first time.

**Distribution**. It is distributed in China (Jilin, Liaoning, Hebei, Ningxia, Gansu, Hunan, Fujian, Guangxi, Yunan, and Hainan) [32], South Korea, Japan, India, Israel, Kyrgyzstan, Tajikistan, Yemen, Russia, Hungary, Estonia, Poland, the Czech Republic, Slovakia, Croatia, Serbia, Montenegro, Moldova, Slovenia, Bulgaria, Romania, Austria, Norway, Finland, Sweden, the United Kingdom, the Netherlands, Germany, France, Italy, Spain, Canada, and the United States [33].

## 4. Discussion

*Scaevola taccada* is an important shrub in the vegetation restoration on islands in the South China Sea [34] but has been severely infested by several insect pests [7]. In this study, we identify three parasitoid species attacking two major pests of *S. taccada*, *H. submarginale,* and *O. scaevolana* on Yongxing Island. According to the host records, these parasitoids are all generalists (i.e., species with a broad host range). Both empirical and theoretical studies have found that island parasitoid communities are biased in favor of generalists when compared with mainland communities [35,36]. As an example of island syndrome [37], island hosts usually suffer higher attack rates by generalist parasitoids [35]. The three parasitoid species we found, especially *D. stantoni* and *E. albitarsis*, show high parasitism rates on their hosts, indicating that these parasitoids are potential biocontrol agents for pests.

A previous study found that *D. stantoni* could be used as a promising candidate for the biological control of the melon borer *Diaphania indica* (Saunders) (Lepidoptera: Crambidae) due to its aggregative response to host density [38]. *Dolichogenidea stantoni* is the only parasitoid species attacking the larvae of *H. submarginale* on Yongxing Island and contributes an average 48.90 (±8.4)% parasitism rate, which is aligned with its performance in infesting *D. indica.* The gregarious reproduction mode of *D. stantoni* should enable it to reproduce large numbers, even when limited host individuals are available. Therefore, *D. stantoni* should be considered a promising biocontrol agent against *H. submarginale*.

The maggots of *O. scaevolana* were infested by two solitary parasitoid species with distinctive different parasitism rates. *Opius biroi* is rare and only occurred in 5.80 (±1.15)% of the surveyed maggots, suggesting that this parasitoid species might not be a promising biocontrol agent against *O. scaevolana.* However, host records indicate that *O. biroi* is a generalist and could infest various leafminer fly species [20]. Since several leafminer species have been found on Yongxing Island [7] (based on personal observation), the effect of *O. biroi* on the populations of other leafminers requires further investigation. With an average parasitism rate of 64.40 (±1.61)%, *E. albitarsis* apparently plays a major role in regulating the population of *O. scaevolana* on Yongxing Island. The biology of *E. albitarsis* largely remains unknown despite its wide range of distribution and diverse host records [32]. Further studies on its biological characteristics are required to explore the possible application of this parasitoid in biocontrol programs.

## 5. Conclusions

Three parasitoid species attack two serious pests of *Scaevola taccada* on Yongxing Island in the South China Sea, viz., *D. stantoni* feeds on the larva of *H. submarginale* and shows a gregarious behavior. *O. biroi* and *E. albitarsis* are both solitary and feed on the larva of *O. scaevolana*. With high parasitism rates on their hosts, *D. stantoni* and *E. albitarsis* have great potential to be used in biocontrol.

## Figures and Tables

**Table 1:** Parasitism and sex ratio of parasitoids of two serious pests feed on *Scaevola taccada*.

Host Species	Parasitoid Species	% Parasitism	% Female Parasitoids
*Herpetogramma submarginale*	*Dolichogenidea stantoni*	48.90 ± 8.40	56.67 ± 2.08
*Ophiomyia scaevolana*	*Opius biroi*	5.80 ± 1.15	67.23 ± 7.51
	*Euderus albitarsis*	64.40 ± 1.61	55.57 ± 6.81

## Data Availability

All data are available in this paper.

## Supplementary Materials

The following supporting information can be downloaded at: https://www.mdpi.com/article/10.3390/insects15120926/s1, Table S1. List of insect pests feed on *Scaevola taccada*; Table S2. List of sequenced species and accession numbers; Table S3. Total number of parasitized larvae of *Herpetogramma submarginale* out of 30 surveyed larvae, emerged parasitoids at three collecting spots; Table S4. Total number of parasitized maggots of *Ophiomyia scaevolana* of 30 leaves, emerged parasitoids at three collecting spots.
