# Supplementary material for: Parasitoids of Insect Pests Feeding on Scaevola taccada (Goodeniaceae) from Yongxing Island in South China Sea
Source: Insects. 2024 Nov 26;15(12):926. doi: 10.3390/insects15120926 (PMC11677017; doi:10.3390/insects15120926)
Supplement: Supplementary file 1 [file insects-15-00926-s001.zip › insects-3324398-supplementary.pdf]

Parasitoids of insect pests feeding on *Scaevola taccada* (Goodeniaceae) from Yongxing Island of South China Sea

Hua-Yan Chen[1,2,3], Cornelis van Achterberg[4], Yang Li[5], Zhen Liu[6], Jun Wang[1,2,3], Shi-Xiao Luo[1,2,3]*

1 Guangdong Provincial Key Laboratory of Applied Botany, South China Botanical Garden, Chinese Academy of Sciences, Guangzhou 510650, China
[2] State Key Laboratory of Plant Diversity and Specialty Crops, South China Botanical Garden, Chinese Academy of Sciences, Guangzhou 510650, China.
[3] South China National Botanical Garden, Guangzhou 510650, China
[4] State Key Laboratory of Rice Biology and Ministry of Agriculture / Key Lab of Agricultural Entomology, Institute of Insect Sciences, Zhejiang University, Hangzhou 310058, China
[5] Sichuan Provincial Key Laboratory for Development and Utilization of Characteristic Horticultural Biological Resources, College of Chemistry and Life Sciences, Chengdu Normal University, Chengdu 611130, China
[6] Zoology Key Laboratory of Hunan Higher Education, College of Life and Environmental Sciences, Hunan University of Arts and Science, Changde 415000, China

*Corresponding author: Shi-Xiao Luo (e-mail: luoshixiao@scbg.ac.cn)

Supplement files

Table S1. List of insect pests feed on *Scaevola taccada*

Order	Family	Species	Damage	Reference
Hemiptera	Monophlebidae	*Icerya seychellarum* (Westwood, 1855)	sucking damage	[1]
Hemiptera	Aphididae	*Aphis gossypii* Glover, 1877	sucking damage	[2]
Thysanoptera	Thripidae	*Thrips unisetifer* Masumoto & Okajima, 2019	sucking damage	[3]
Diptera	Agromyzidae	*Liriomyza sativae* Blanchard, 1938	leaf mines	[2]
Diptera	Agromyzidae	*Ophiomyia cornuta* Meijere, 1910	leaf mines	[4]
Diptera	Agromyzidae	*Ophiomyia scaevolana* Shiao & Wu, 1996	leaf mines	[5]
Hymenoptera	Argidae	*Arge geei* Rohwer, 1912	chewed leaves	[2]
Coleoptera	Chrysomelidae	*Phaedon brassicae* Baly, 1874	chewed leaves	[2]
Coleoptera	Chrysomelidae	*Colaphellus bowringi* (Baly, 1865)	chewed leaves	[2]
Coleoptera	Chrysomelidae	*Phyllotreta striolata* (Fabricius, 1803)	skeletonized leaves	[2]

References

1. Hill, M.G. Susceptibility of *Scaevola taccada* (Gaertn.) Roxb. Bushes to attack by the coccid *Icerya seychellarum* Westwood: the effects of leaf loss. *Ecol. Entomol.* **1980**, 5, 345–352.
2. Chen, Q.; Liang, X.; Wu, CL.; Chen, Q. Pest survey and safety assessment on five Islands of Yongle Archipelago. *Chinese Journal of Tropical Crops*, **2020**, 41, 148–156. (Text in Chinese)

3. Masumoto, M.; Okajima, S. Three new species of the genus *Thrips* (Thysanoptera, Thripidae) in Japan. *Zootaxa* **2019**, 4614, 575–584.
4. Wijesekara, A. Synopsis of the Agromyzidae (Diptera) of Sri Lanka. *Cey. J. Sci. (Bio. Sci.)* **2002**, 29, 41–62.
5. Shiao, S. F. and Wu, W.J. Four new agromyzid species from Taiwan (Diptera: Agromyzidae). *Trans. Am. Entomol. Soc.* **1996**, 122, 213–226

Table S2. List of sequenced species and accession numbers.

Code	Species	Sex	Host	GenBank Accession No.
SCBG-E0004443	*Dolichogenidea stantoni*	female	*Herpetogramma submarginale*	PQ530501
SCBG-E0004444	*Dolichogenidea stantoni*	male	*Herpetogramma submarginale*	PQ530502
SCBG-E0004447	*Opius biroi*	female	*Ophiomyia scaevolana*	PQ530503
SCBG-E0004448	*Opius biroi*	male	*Ophiomyia scaevolana*	PQ530504
SCBG-E0004449	*Euderus albitarsis*	female	*Ophiomyia scaevolana*	PQ530505
SCBG-E0004450	*Euderus albitarsis*	male	*Ophiomyia scaevolana*	PQ530506

Table S3. Total number of parasitized larvae of *Herpetogramma submarginale* out of 30 surveyed larvae, emerged parasitoids at three collecting spots

Spot	No. of parasitized larvae	No. of parasitoids	No. of female parasitoids
1	15	150	84
2	12	105	62
3	17	156	86

Table S4. Total number of parasitized maggots of *Ophiomyia scaevolana* of 30 leaves, emerged parasitoids at three collecting spots

		No. of parasitoids		No. of female parasitoids	
spot	No. of parasitized maggots (total No. of maggots)	*Opius biroi*	*Euderus albitarsis*	*Opius biroi*	*Euderus albitarsis*
1	44 (65)	3	41	2	26
2	51 (72)	5	46	3	24
3	49 (68)	4	45	3	23
